# Five-year changes in soil organic carbon and total nitrogen in coastal wetlands affected by flow-sediment regulation in a Chinese delta

**DOI:** 10.1038/srep21137

**Published:** 2016-02-16

**Authors:** Junjing Wang, Junhong Bai, Qingqing Zhao, Qiongqiong Lu, Zhijian Xia

**Affiliations:** 1 State Key Laboratory of Water Environment Simulation, School of Environment, Beijing Normal University, Beijing 100875, China

## Abstract

Changes in the sources and sinks of soil organic carbon (SOC) and total nitrogen (TN) in wetland soils as indicators of soil quality and climate change have received attention worldwide. Soil samples were collected in 2007 and 2012 in the coastal wetlands of the Yellow River Delta and the SOC and TN were determined to investigate a five-year change in their content and stock in these wetlands as affected by flow-sediment regulation. Our results revealed that the soils in 2007 exhibited greater electrical conductivities, SOC content and density, and ammonium nitrogen (NH_4_^+^-N) levels in the top 10 cm soils (*p* < 0.05) compared with the soils in 2012. In general, the SOC and TN contents decreased with increasing soil depth. However, the highest ratios of soil organic carbon and total nitrogen (molar C/N ratios) were observed in the 30–40 cm soil layer. A significant SOC loss occurred (*p* < 0.05) in top 10 cm soils, but only a small change in SOC in the top 50 cm soils. Comparatively, TN levels did not show significant differences in the study period.

As one important pool of soil organic matter in natural wetland ecosystems, wetland soils serve as sources, sinks and transfers of nutrients and chemical pollutants^1^,^2^,^3^. Soil carbon and nitrogen are the important components of sustainable soil fertility and productivity and can substantially affect climate change through carbon and nitrogen emissions (including CO_2_, CH_4_ and N_2_O). However, anthropogenic activities, including deforestation, biomass burning and land use changes^4^,^5^ might alter soil C and N levels^6^. Mitsch and Gosselink^7^ reported that any variations in the distributions and abundances of soil C and N exert important effects on the carbon and nitrogen cycles at regional or global scales. Therefore, the accurate estimations of soil organic carbon (SOC) and total nitrogen (TN) are essential for the detection of potential carbon and nitrogen sequestrations and emissions in wetlands^8^. Some researchers have focused on SOC and TN contents and stocks in different ecosystems including wetlands^9^,^10^,^11^. It is estimated that 20–30% of the earth’s soil carbon pool of 2,500 Pg is stored in wetlands because wetlands provide an optimum natural environment for the sequestration and long-term storage of carbon^12^. However, the dynamics of carbon and nitrogen pools in wetland soils are still poorly understood, particularly in coastal wetlands.

Although salt marsh wetlands are blue carbon sinks that will contain carbon accumulations for millennia^14^, they have been seriously lost or degraded due to human activities^4^,^6^,^13^. Among the effects of major human activities on riparian and estuarine ecosystems, large dams and reservoirs on rivers or streams have remarkably affected the wetland ecosystems of the lower reaches and altered many ecological processes, structures and functions^15^,^16^,^17^. Magilligan and Nislow^15^ documented the types, magnitudes and directions of hydrological shifts and climatic regimes due to the impoundment of the National Inventory of Dams. Barros *et al.*^18^ also concluded that future carbon emissions would be highly dependent on the locations of reservoirs because the reservoirs contain large stores of organic carbon transported from terrestrial ecosystems which might enhance greenhouse gas emissions.

Changes in the hydrological regime of wetlands can have substantial effects on soil properties, particularly carbon and nitrogen accumulation and release due to alterations in their chemical forms and spatial movements^19^,^20^. The rewetting of dried soils could stimulate denitrification, whereas N mineralization didn’t significantly decrease in wetland soils^21^. However, multiple drying and wetting cycles would enhance the decomposition of SOC^22^. Additionally, Wang *et al.*^23^ reported that freshwater restoration could elevate SOC and TN contents in degraded coastal wetlands. Although Bai *et al.*^9^ and Zhao *et al.*^24^ investigated the seasonal changes in SOC and TN contents and stocks in coastal wetlands and the influence of flow-sediment regulation on these parameters, little information is available about the medium-term (e.g., five-year) influences of flow-sediment regulation on changes in SOC and TN in downstream wetlands. Thus, an improved understanding of the quantitative medium-term dynamic changes in soil carbon and nitrogen will contribute to the evaluation and identification of sources and sinks of soil carbon and nitrogen and guide the flow-sediment regulation.

The Yellow River Delta is a coastal wetland with a heavy burden of anthropogenic activities, which has been severely affected by sediment deposition and land-ocean interactions relative to other river deltas^4^,^25^. In order to control soil erosion and degradation and improve soil productivity, huge amounts of efforts have been expended on the Yellow River Delta. Flow-sediment regulations of the upstream Xiaolangdi reservoir have been implemented in late June and early July of each year since 2002^26^. Thus, in the last decade, the freshwater input from flow-sediment regulation has been shown to be an effective method for reconstructing or restoring the degraded estuarine wetlands^27^. Moreover, the freshwater input has significantly enhanced SOC accumulation in the degraded coastal wetlands due to the restoration of wetland vegetation^23^. Yu *et al.*^4^ reported a slight increase in SOC stock in the top 30 cm soils from 2000 to 2009 based on land use changes using a method of replacing time with space, but the estimated results exhibited a substantial uncertainty due to the lack of reference SOC data from 2000. Therefore, an investigation of the accurate medium-term dynamic changes in SOC and TN stocks are still needed in these coastal wetlands to identify the effects of flow-sediment regulation of the upstream reservoir. The primary objectives of the present study were: 1) to investigate the five-year (from 2007 to 2012) changes in the SOC and TN contents and stocks in the coastal wetlands in the Yellow River Delta as affected by flow-sediment regulation, and (2) to identify the key factors influencing the soil carbon and nitrogen levels.

## Results and Discussion

### Soil characterization in 2007 and 2012

Selected physical-chemical properties of the top 10 cm soils from the coastal wetlands in the Yellow River Delta in 2007 and 2012 are summarized in Table 1. No significant differences in soil pH, bulk density (BD) and soil water content (SWC) in the top 10 cm soils were observed between 2007 and 2012 due to the counterbalance of consistent flow and sediment variations. However, the top 10 cm soils contained lower SOCDs in 2012 than in 2007, which was associated with lower SOC contents in 2012 (*p* < 0.05; Table 1). The electrical conductivity (EC) and NH_4_^+^-N contents in the top 10 cm soils were significantly higher in 2007 than those in 2012 (*p* < 0.05), but no significant differences in the TN, TN density (TND) and nitrate nitrogen (NO_3_^−^-N) contents were observed (*p* > 0.05). Higher nitrogen deposition levels, i.e., ~2264.24  mg /m^2^ in this region^28^,^29^, might have supplemented the nitrogen loss during the study period. Additionally, a decrease in NH_4_^+^-N content from 2007 to 2012 (*p* < 0.05) might be explained by the fact that plant uptake or ammonia volatilization under medium alkaline environment in this region as well as the conversion to NO_3_^−^-N through nitrification, although nitrate could be leached into deeper soils^30^,^31^.

### SOC content and stock

**Figure 1:**
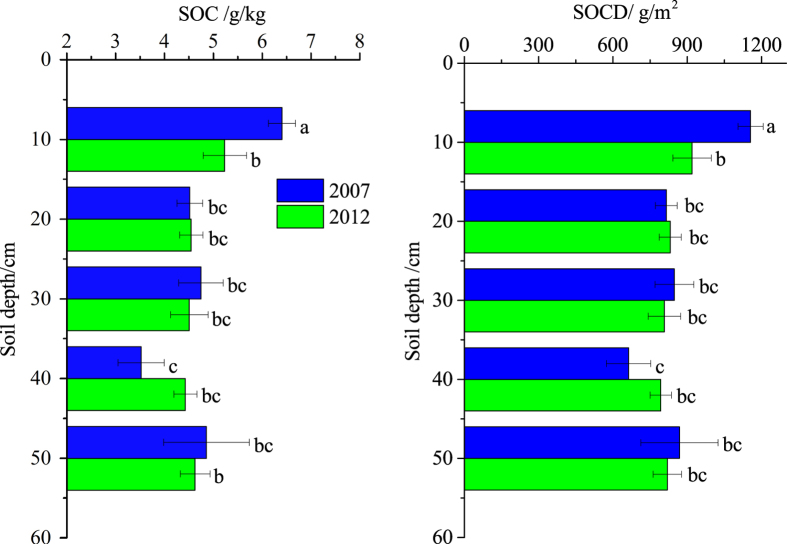
Depth distributions of SOC content and SOCD in 2007 and 2012. ^abc^Different letters at the depths indicate significant differences at *p* < 0.05. The error bars represent the standard deviations.

The SOC content and SOCD generally exhibited decreasing tendencies with depth along the soil profiles at each sampling site (Fig. 1). The top 10 cm soils contained significantly higher SOC content (6.41 ± 0.28 g/kg) and SOCD (1155.80 ± 50.46 g/m^2^) than deeper soils (SOC, 3.52–4.86 g/kg; SOCD, 663.07–868.46 g/m^2^) in 2007 (*p* < 0.05). Although no significant differences were observed (*p* > 0.05), the SOC content (5.23 ± 0.44 g/kg) and SOCD (919.67 ± 77.58 g/m^2^) in the top 10  cm soils were slightly higher than those in deeper soils (SOC, 4.43–4.63 g/kg; SOCD, 793.02–831.20 g/m^2^) in 2012. The contributions of SOCD in the top 10 cm soils accounted for 26.57% and 22.05% in 2007 and 2012, respectively. With the exception of a significant SOC loss in the top 10 cm soils, the SOC and SOCD exhibited only small changes in deeper soils (i.e., 10–50 cm soils). Plant cycling could explain the higher levels of SOC and SOCD in the top soils^32^. Qin *et al.*^33^ also found that carbon inputs from plant roots and plant residues were often accumulated in the surface soil. Compared with surface soils, only a small decrease in SOCD (4.12%) in the top 50 cm soils occurred from 2007 to 2012 in this study, although Yu *et al.*^4^ observed a slight increase in SOC storage in the top 30 cm soils in the entire Yellow River Delta from 2000 to 2009. This finding indicated that there was a five-year effect on SOC content and stock in the top 10 cm soils in coastal wetlands. This might be associated with the long-term soil organic matter (SOM) breakdown due to freshwater input, which has been affected by the flow-sediment regulation since 2002^34^. The freshwater input could increase SOC loss through changing soil salinity since soil salinity was strong negatively correlated with microbial biomass carbon and soil organic matter content^35^,^36^. Chambers *et al.*^37^ also found that CO_2_ flux was higher in the freshwater marsh than brackish and salt marshes in a 53-day laboratory experiment. Moreover, the drying and wetting cycles dominated by the overbank flow from the flow-sediment regulation might increase the SOC loss. Xu *et al.*^22^ reported that multiple drying and wetting cycles would enhance the decomposition of SOC in paddy soils. Aerts and Ludwig^38^ observed that periodical changes in aeration or the water table could increase cumulative C mineralization rates in laboratory columns of peatland soils. Additionally, small additional dissolve organic carbon inputs have been observed from the upper organic horizons to the deeper mineral soils due to drying and wetting cycles^39^.

### TN content and TND

**Figure 2:**
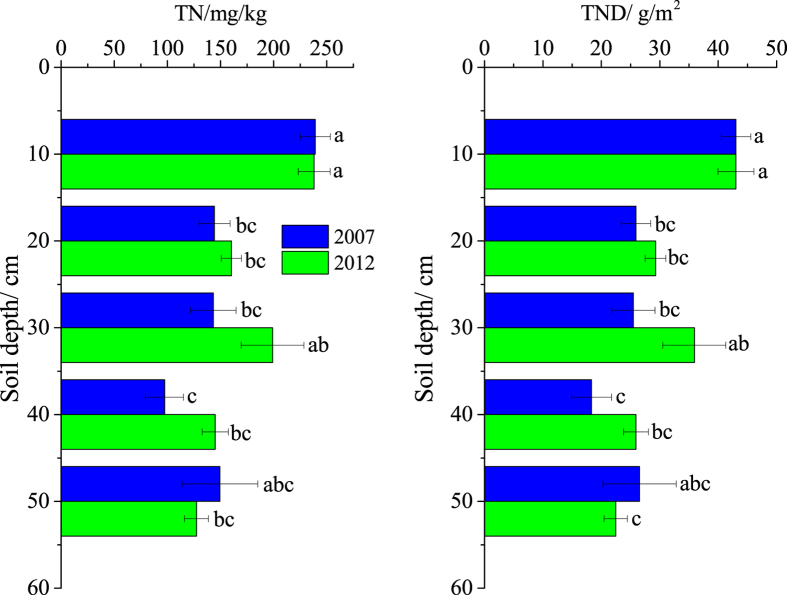
Depth distributions of the TN content and TNDs in 2007 and 2012. ^abc^Different letters at the depths indicate significant differences at *p* < 0.05. The error bars represent the standard deviations.

The distributions of TN content and TND in the two sampling periods of 2007 and 2012 are shown in Fig. 2. Similar to the SOC content and SOCD, the TN content and TND generally exhibited decreasing tendencies with depth, with the exception of accumulations at the 20–30 cm soil depth in 2012. This observation agrees with the previous results^9^ in which the TN content was observed to decrease with increasing depth, and TN accumulation was observed in the deeper soils of coastal wetlands in the Yellow River Delta. Wang *et al.*^40^ also observed a decrease trend in soil nitrogen sequestration with soil depth. The top 10 cm soils contained significantly higher TN level and TNDs compared to the deeper soils (with the exception of the 20–30 cm soil depth in 2012) in both sampling periods (*p* < 0.05) that accounted for 27.48–30.89% of the total TND in the whole soil profiles. This is attributed to the fact that nitrogen can return to surface soils through plant cycling^32^. Moreover, greater nitrogen deposition in this region would contribute to elevated N levels in the surface soils^4^. Nitrogen accumulation at the 20–30 cm soil depth might be associated with the leaching of dissolved organic nitrogen and inorganic nitrogen because plants and microbes cannot prevent dissolved organic matter losses^41^. Ammonia nitrogen also exhibited a substantial accumulation at the 20–30 cm soil depth, but only a small decrease in nitrate nitrogen was observed with soil depth. These findings could be explained by leaching and the rapid transformation rates of nitrate nitrogen in surface soils (0–10 cm)^42^.

Water table fluctuation is the primary determinant of N dynamics and their end products, which controls the aerobic/anaerobic conditions in the soil^43^. Venterink *et al.*^21^ also found that repeated drying and wetting increased the cumulative NH_4_^+^-N but reduced NO_3_^−^-N, which is indicative of a reduction in net nitrification in deeper soils. However, no significant changes in the TN contents or TNDs were observed along the soil profiles between 2007 and 2012 (*p* > 0.05). Similarly, the TND values in the top 50 cm soils also did not significantly change from 2007 to 2012 although an increment was observed (*p* > 0.05). Therefore, there were no significant five-year effects on the TN contents or TNDs in the coastal wetlands.

### Soil molar C/N ratios

**Figure 3:**
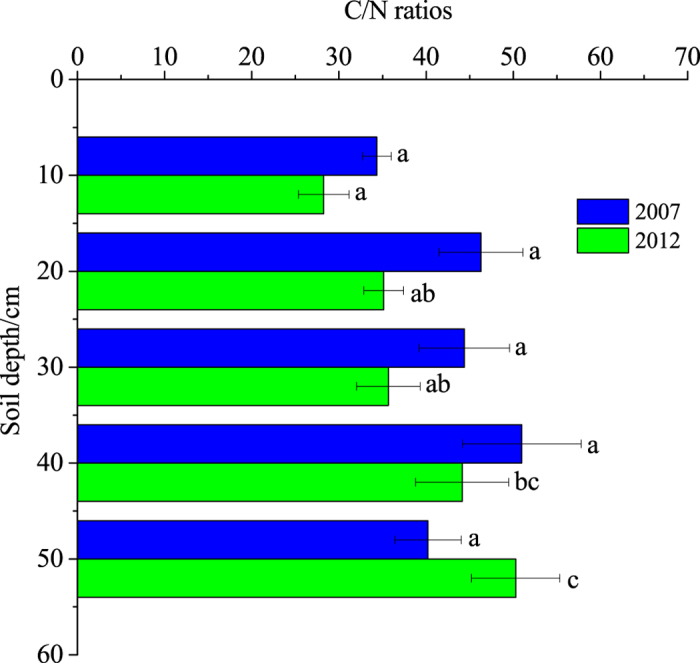
Depth distributions of the C/N ratios in 2007 and 2012. ^abc^Different letters at the depths indicate significant differences at *p* < 0.05. The error bars represent the standard deviations.

The molar C/N ratios are a good indicator and useful tool for identifying the terrestrial-based or marine-based sources of soil organic matter and nitrogen limitation of plants in terrestrial ecosystems^44^,^45^. Figure 3 illustrates the variations in soil C/N ratios with soil depth in 2007 and 2012. Generally, the soil C/N ratios in 2012 increased significantly with soil depths (*p* < 0.05) and the values ranged from 28.25 to 50.27. Comparatively, the highest C/N ratio (50.96) was observed at the 30–40 cm soil depth in 2007, no significant differences were found between soil depths (*p* > 0.05). However, Lu *et al.*^46^ found that C/N ratios decrease gradually with increasing soil depth in reed wetlands. This might be associated with the freshwater input from the flow-sediment regulation, which limited microbial activity in the deeper soils^47^ and led to a slow decomposition rate of organic matter. Additionally, the C/N ratio was lowest in the 0–10 cm soils (*p* < 0.05) in both years. The average soil C/N ratios in the top 50 cm soils were 43.24 in 2007 and 38.69 in 2012. Soil C/N ratios as the mineralization potential index were greater than 25 to 30 which is associated with N contents limit decomposition^23^,^48^. The C/N ratios remained > 25 implies that the SOM in this region was primarily from terrestrial sources (terrestrial-based organic matter has a C/N ratios greater than 20)^47^ and that the SOM decomposition would be limited by nitrogen availability due to higher C/N ratios (exceeding 25)^23^. A slight decrease in the C/N ratios was observed from 2007 to 2012, which might be an indication of more decomposed residues because less mineralization occurred in 2007 than in 2012 (*p* < 0.05; Table 1).

### Relationships between carbon, nitrogen and selected soil properties

The results of Pearson correlation analysis indicated that there were significant relationships among the SOC, SOCD, TN, and TND in the top 20 cm soils in both periods (*p* < 0.01; Table 2). Consistent changes in the SOC and TN levels in many different wetlands have been reported by the majority of researchers^4^,^9^,^11^. Significant positive correlations between pH values and soil C and N indicated that higher pH ranges (i.e., medium alkaline) prohibited soil C and N decomposition through influencing microbial activity^48^. Soil moisture might be a dominant factor in the determination of SOM decomposition^49^,^50^. The SOC, TN, and TND exhibited significant correlations with the soil moisture in this study, which indicates that higher soil moisture in coastal wetlands contributed to C and N accumulation due to anaerobic conditions^21^,^43^,^51^. Soil bulk density exhibited a significant negative correlation with SOC (*p* < 0.01), which is consistent with the results reported by Addis *et al.*^52^ because bulk density is generally negatively correlated with soil moisture. Additionally, NH_4_^+^-N was significantly correlated with SOC, SOCD, TN and TND (*p* < 0.01), which implies that organic nitrogen mineralization was dominated by the C and N contents in the soil^53^. The negative correlations of TN and TND with the C/N ratios demonstrated that the higher C/N ratios in the coastal wetlands enhanced nitrogen loss because SOC could act as an electron donor in the denitrification of NO_3_^−^-N^3^.

**Figure 4:**
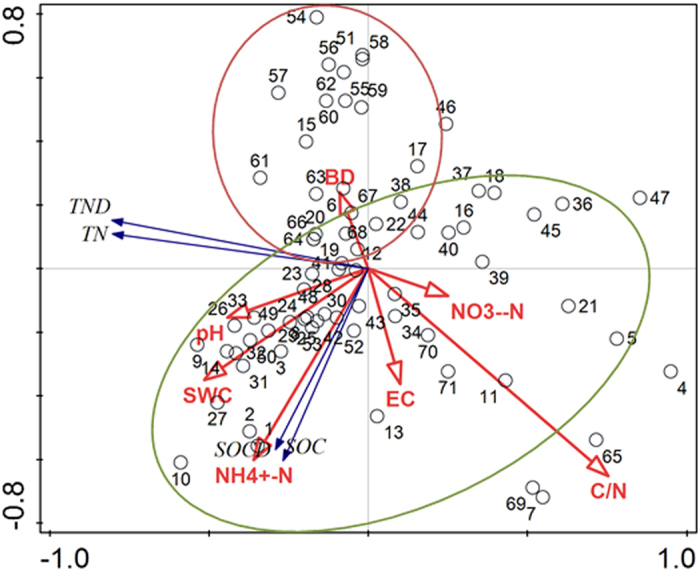
Bioplot of RDA for the relationships between SOC and TN contents and stocks and selected soil properties in surface soils in 2007 (Sites1–44) and 2012 (Sites 45–71).

The relationships of the soil carbon and nitrogen (i.e., the SOC, SOCD, TN and TND) with the selected soil factors at these sampling sites are summarized in a bioplot of RDA of the two sampling periods (Fig. 4). In this biplot diagram, the blue arrows represent the soil carbon and nitrogen, and the red arrows represent the influencing factors. The circles indicate the sampling sites, and the lengths of the arrows represent the strengths of the influences of the selected soil parameters on the SOC and TN. The first two axes of the RDA explained a high proportion of the variance (i.e., 42.55% and 14.34% for axes 1 and 2, respectively). The results also revealed the relationship between the sampling sites, which were clustered in the RDA diagram. The samples collected in 2007 (S1–S44) were closely distributed along the first axis, which reflected the significant effects of pH and SWC. The samples collected in 2012 (S45–S71) were closely distributed along the second axis, which indicated that the soil C and N were dominantly affected by the soil C/N ratio and NH_4_^+^-N.

## Conclusions

We investigated the temporal changes in soil organic carbon and total nitrogen in the riparian wetlands of the Yellow River Delta from 2007 to 2012. The results clearly illustrated a negative effect of flow-sediment regulation on the soil C content in the surface soil over the five- year period. A significant SOC loss of 20.43% in the top 10 cm soils were observed from 2007 to 2012, however, only a small change in SOC stock in the top 50 cm soils were observed, indicating great effects of environmental factors on surface soils. Comparatively, no significant changes in TN content and stock were observed in the study period. The SOC and TN contents and stocks generally decreased along the soil profiles, and they were significantly higher in the top 10 cm soils than the deeper soils with the exception of the SOC and SOCD in 2012. The soil pH and SWC exhibited the strongest effects on soil C and N in 2007, whereas the soil C/N ratios and NH_4_^+^-N were the dominant influencing factors in 2012. The findings of this study will contribute to guiding freshwater restoration and flow-sediment regulation to enhance the ecological functions of carbon and nitrogen pools and provide basic data regarding global C and N cycles. However, further studies involving the dynamic monitoring of soil C and N are still needed to determine their long-term changing patterns in coastal wetlands that are affected by freshwater input and seawater intrusion.

## Materials and Methods

### Study area

The study area is located in the Yellow River Delta (N37°45′57.8″, E119°11′15.2″) of the eastern part of Shandong Province on the southern bank of the Bohai Gulf. The Yellow River Delta is one of the most extensive and youngest regions, and it is also the fastest growing wetland among large river deltas worldwide. This region has a warm-temperate and continental monsoon climate with distinct seasons that include a rainy summer. The annual mean temperature is 11.7–12.9 °C. The annual mean precipitation ranges from 550 to 640 mm, and the annual mean evaporation is 1962 mm^54^, which causes serious drought disasters. The groundwater table in this region is shallow with an average depth of 1.14 m, and the groundwater contains TDS in quantities that vary from 0.54 to 97.56 g/L (data from the Chinese Institute for Hydrogeology and Engineering Geology Survey, CIHEGS, 1986). The dominate vegetation in coastal wetlands in the Yellow River Delta includes herbaceous plants (*Phragmites australis* and *Suaeda salsa*) and shrubs (*Tamarix chinensis*)^9^. Because the frequencies of drying or ephemeral stream flows have been increasing in the Yellow River since the early 1970s, the Xiaolangdi Reservoir began storing water in late 1999, which has substantially controlled the flow of the lower Yellow River; indeed this water storage represents the hydrological beginning of the lower Yellow River^55^. A flow-sediment regulation regime has been implemented in the upstream Xiaolangdi Reservoir since 2002 by the Yellow River Conservancy Committee to control the discharges of water and sediment.

### Sample collection and analysis

The coastal wetlands affected by the flow-sediment regulation were selected in the Yellow River Delta. In total, 71 sampling sites were collected to depths of 50 cm in coastal wetlands from May to August in the study periods, which included 44 sampling sites in 2007 and 27 sampling sites in 2012. The soil profiles with three replicates for each sampling site were sectioned into five depths at 10 cm intervals and mixed with the same soil layers to form composite samples. Additional soil cores (4.8 cm diameters) from each soil layer of each sampling site were collected to determine the soil moistures and bulk densities. The studied wetlands were consistently affected by the flow-sediment regulation that has been implemented since 2002. Soil samples were collected in 2007 (affected by the flow-sediment regulation for five years) and in 2012 (affected by the flow-sediment regulation for ten years). Thus the five-year changes in SOC and TN were identified by comparisons of the two sampling years. The soil samples were obtained and stored at 4 °C in sealed plastic bags to limit microorganism activity for the determination of the soil carbon and nitrogen. All of the soil samples were air-dried at room temperature for three weeks, and coarse debris, roots and stones were removed. Then all soil samples were crushed to pass through a 0.149-mm stainless steel mesh and stored for the determination of chemical analyses.

The soil pH were measured using a Hach pH meter (Hach Company, Loveland, CO, USA), and the EC values were measured using a conductivity meter (Mettler Toledo, USA) in the supernatants of 1:5 soil:water mixtures. The SWC and BD were determined by oven-drying at 105 °C for 24 h. The SOC levels were determined using dichromate oxidation^56^. The TN levels were measured in duplicate using dry combustion with an Elementar Vario C/N Analyzer. Additionally, the NO_3_^−^-N and NH_4_^+^-N levels were analyzed using AA3 automated flow injection analysis (Bran + Luebbe GmbH, Germany).

The SOC densities and TN densities at certain soil layer of each sampling site were calculated using Eqs. (1) and (2), respectively.


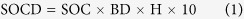



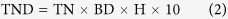


where SOCD and TND are the soil organic carbon density (g/m^2^) and the total nitrogen density (g/cm^2^), respectively, SOC is the soil organic carbon content (g/kg), TN is the total nitrogen content (g/kg), BD is the soil bulk density (g/m^3^), and H is the thickness of the soil layer (cm). SOCD in the whole soil profile is the sum of SOCD at each soil layer.

As for the calculation of C/N ratios, the contents of soil organic carbon and total nitrogen were transformed to a unit of mol/kg, thus the C/N ratios were calculated as molar ratio rather than mass ratios.

### Statistical analyses and graphing

The statistical analyses were conducted using SPSS 16.0 software package. One-way ANOVA was performed to identify the significant differences in the mean values of the soil properties between the sampling sites, the soil depths and the sampling years. Differences were considered significant at the 0.05 level based on least significance difference (LSD) tests. Redundancy analysis (RDA) was conducted using the Canoco 5.0 software package (Microcomputer Power, Ithaca, USA) to identify the main factors that influenced SOC and TN contents and stocks in the top 10 cm soils between 2007 and 2012. The figures were created using the Origin 8.0 (Origin Labs Corporation, Northampton, Massachusetts, United States) and Canoco5.0 software packages.

## Additional Information

**How to cite this article**: Wang, J. *et al.* Five-year changes in soil organic carbon and total nitrogen in coastal wetlands affected by flow-sediment regulation in a Chinese delta. *Sci. Rep.* **6**, 21137; doi: 10.1038/srep21137 (2016).

## Figures and Tables

**Table 1:**
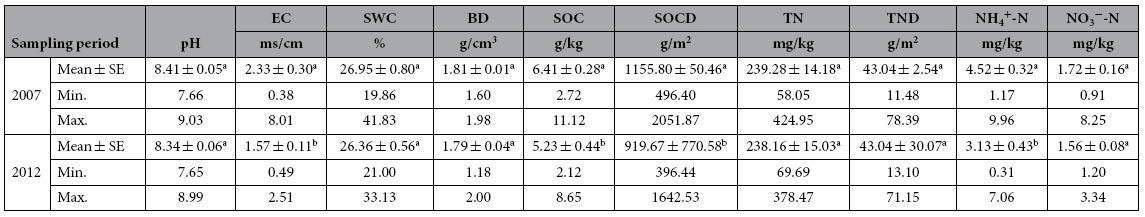
Mean values of selected soil properties in the top 10 cm soil in 2007 and 2012.

^a,b^The different letters represent significant differences between 2007 and 2012.

**Table 2:** Correlation analysis of the soil nutrients and soil environmental parameters.

	SOC	SOCD	TN	TND	C/N ratios	pH	EC	SWC	BD	NH_4_^+^-N	NO_3_^−^-N
SOC	1.000										
SOCD	0.560**	1.000									
TN	0.731**	0.382**	1.000								
TND	0.701**	0.371**	0.992**	1.000							
C/N ratios	−0.139	0.002	−0.617**	−0.624**	1.000						
pH	0.272**	0.227**	0.362**	0.353**	−0.231**	1.000					
EC	0.094	0.200*	−0.107	−0.092	0.255**	−0.334**	1.000				
SWC	0.369**	0.164	0.415**	0.392**	−0.229**	0.200*	−0.162	1.000			
BD	−0.267**	−0.022	−0.099	0.023	−0.006	−0.044	0.104	−0.179*	1.000		
NH_4_^+^_-_N	0.410**	0.355**	0.260**	0.287**	−0.022	0.058	0.211*	0.094	0.240**	1.000	
NO_3_^−^-N	0.014	0.061	−0.096	−0.106	0.147	0.010	0.298**	0.033	−0.025	−0.058	1.000

^*^Significant correlation at *p* < 0.05; **Significant correlation at *p* < 0.01.
